# Are Medicare Funded Multidisciplinary Care Policies Being Claimed in accordance to Rehabilitation Needs in Patients with Stroke?

**DOI:** 10.31083/j.rcm2309318

**Published:** 2022-09-14

**Authors:** David A Snowdon, David Ung, Taya A Collyer, Natasha A Lannin, Monique F Kilkenny, Amanda G. Thrift, Vijaya Sundararajan, Dominique A Cadilhac, Nadine E Andrew

**Affiliations:** ^1^Department of Medicine, Peninsula Clinical School, Central Clinical School, and National Centre for Healthy Ageing, Monash University, 3199 Frankston, Australia; ^2^Department of Neuroscience, Central Clinical School, Monash University, 3004 Melbourne, Australia; ^3^Occupational Therapy Department, Alfred Health, 3004 Melbourne, Australia; ^4^Stroke and Ageing Research, Department of Medicine, School of Clinical Sciences at Monash Health, Monash University, 3168 Clayton, Australia; ^5^Florey Institute of Neuroscience and Mental Health, University of Melbourne, 3084 Heidelberg, Australia; ^6^Department of Public Health, School of Psychology and Public Health, La Trobe University, 3086 Bundoora, Australia; ^7^Department of Medicine, St Vincent’s Hospital, Melbourne Medical School, University of Melbourne, 3065 Fitzroy, Australia

**Keywords:** stroke, primary health care, referral and consultation, epidemiology, secondary prevention

## Abstract

**Background:**

Australian Primary Care Practitioners are incentivised through Medicare funded policies to provide chronic disease management and facilitate multidisciplinary care. Little is known about how these policies are claimed in the long-term management of stroke. The objective of this study was to describe the use of funded primary care policies for people with stroke by impairment status.

**Methods:**

Linked Australian Stroke Clinical Registry (2010–2014) and Medicare data from adults with 90–180 days post-stroke EQ-5D health status survey data and admitted to one of 26 participating Australian hospitals were analysed. Medicare item claims for Primary Care Practitioner led chronic disease management and multidisciplinary care coordination policies, during the 18 months following stroke are described. Registrants were classified into impairment groups using their EQ-5D dimension responses through Latent Class Analysis. Associations between impairment and use of relevant primary care policies were explored using multivariable regression.

**Results:**

5432 registrants were included (median age 74 years, 44% female, 86% ischaemic), 39% had a chronic disease management claim and 39% a multidisciplinary care coordination claim. Three latent classes emerged representing minimal, moderate and severe impairment. Compared to minimal, those with severe impairment were least likely to receive chronic disease management (adjusted Odds Ratio (aOR): 0.61, 95% Confidence Interval (CI): 0.49, 0.75) but were most likely to receive multidisciplinary care coordination. Podiatry was the commonest allied health service prescribed, regardless of impairment.

**Conclusions:**

Less than half of people living with stroke had a claim for primary care initiated chronic disease management, with mixed access for those with severe impairments.

## 1. Introduction

Over 50% of Australians have at least one chronic disease. Stroke is a complex and costly disease. People living with stroke, require management of risk factors to prevent future cardiovascular events, and often have other chronic diseases and impairment. Of concern, people living with stroke report long-term unmet needs, particularly beyond 6 months post-stroke [1, 2].

These long-term unmet needs relate to a wide range of impairments in body function (e.g., fatigue, memory/concentration, speech) and activity and participation limitation (e.g., mobility, leisure, employment), which are associated with lower quality of life and increased risk of cardiovascular disease due to lifestyle impacts [3, 4]. As such, it is recommended that people living with stroke receive annual reviews to identify unmet needs and referral to a multi-disciplinary healthcare team to address identified problems [5, 6]. However, this care can be difficult to co-ordinate due to organisational and budgetary boundaries between hospital and community care which negatively impact transfer of care and provision of community based services [5].

Since 1999, the Australian government has invested substantial funds for policies that promote enhanced models of primary care. Australian Primary Care Practitioners, also known as General Practitioners, are incentivised through Medicare funded policies to provide chronic disease management and coordinate multidisciplinary care planning. These items are targeted at facilitating care, in accordance with clinical guidelines, using a coordinated, multi-disciplinary approach and promoting self-management of chronic disease [7]. Additional funding beyond that available for a standard consultation is provided via specific Medicare claim items. Funding items are also provided for reviewing these plans every three months, ensuring that care is responsive to patients’ changing needs [8]. Those requiring multi-disciplinary team-based support can also access Medicare subsidised allied health services, which may help address their long-term unmet rehabilitation and secondary prevention needs. Despite serving an important function, Primary Care Practitioner use of these guidelines and utilisation of these care types in a large, nationally representative sample, with reference to the impairment profile of survivors, is currently unknown.

We aimed to describe the use of Medicare funded chronic disease management, and multidisciplinary care coordination policies for people with stroke living in the community and how this varied by impairment status.

## 2. Materials and Methods

This is a retrospective, observational, cohort-based data linkage study. The cohort was derived from the Australian Stroke Clinical Registry (AuSCR), and included adults aged ≥18 years admitted to one of 26 participating hospitals from five Australian states between April 1, 2010 and December 31, 2014. Patient-level registry data were linked to Medicare and pharmaceutical claims data to June 31, 2016 [9, 10]. For this analysis we restricted the cohort to those with stroke (i.e., excluding those with Transient Ischaemic Attack [TIA]) who completed AuSCR six-month follow-up questionnaires and were living in the community at the time of completion. We excluded those living in residential aged care as they are not eligible for these items.

Ethics approvals for this study were obtained from Monash University (#7864) and the Australian Institute of Health and Welfare (#EO2017/1/346). Approvals were also received from the AuSCR Research Task Group, the AuSCR Steering Committee, and the Queensland Public Health Act. The AuSCR holds approval for collecting data via an opt-out model of consent, with a waiver of consent for those who died in hospital.

### 2.1 Healthcare in the Australian Setting

Australia has universal healthcare whereby all citizens and permanent residents are eligible for basic healthcare that includes services provided by Primary Care Practitioners. Unlike standard claims, the chronic disease Medicare funded policies have regulatory guidelines [11]. To be eligible for a chronic disease management plan, patients must have a chronic or terminal condition, defined as one that has been, or is likely to be, present for at least 6 months [8]. To be eligible for multidisciplinary care coordination, patients must have a chronic or terminal condition that requires the Primary Care Practitioners to coordinate care with at least two collaborating health professionals, each of whom will provide a different kind of treatment or service, one of whom may be another medical professional [8]. Although eligibility for these two schemes overlap, Primary Care Practitioners can provide them independently. A multidisciplinary care coordination plan also allows access to 13 different allied health professions at private clinics with costs heavily subsidised by Medicare for the first five visits in a calendar year. While not all patients will use Medicare, to access services, a multidisciplinary care coordination plan can still be used to compensate the Primary Care Practitioners for time taken to coordinate patient care regardless of the funding model [8]. For example to coordinate rehabilitation care or dietetic advice received in a hospital outpatient setting.

### 2.2 Datasets

*The AuSCR* is a prospective national clinical quality registry used to monitor quality of acute stroke care in Australian hospitals (http://www.auscr.com.au). Data are collected on all patients with clinical diagnosis of stroke or TIA admitted to participating hospitals [12]. Survival status is determined through annual linkages with the National Death Index. Eligible registrants are contacted at 90–180 days following stroke admission to complete a follow-up questionnaire that includes the 5-Dimension, 3-level, European Quality of Life Scale (EQ-5D-3L) questionnaire and information on where they are living. The EQ-5D-3L includes questions on five functional and health domains (mobility, self-care, usual activity, anxiety/depression, pain/discomfort) scored over three levels (none, moderate, severe). It also contains a visual analogue scale where respondents are asked to score their health state from zero (worst imaginable health) to 100 (best imaginable health).

*Medicare Benefits Schedule* (*Medicare*) contains all transactional claims related to health services subsidised by the Australian Commonwealth Government such as primary care and medical specialist visits, pathology and radiology. Items are also available to provide financial incentives for Primary Care Practitioners to provide enhanced models of primary care and visits to allied health practitioners.

*Pharmaceutical Dispensing Claims* contain details of all subsidised prescription medications dispensed to Australian residents. Most patients (>90%) in this study were eligible for subsidised medications at the time of their stroke and are therefore included in this database [10].

### 2.3 Variable definitions

Claims for chronic disease management policies, multidisciplinary care coordination, specialists and allied health encounters were identified using the Medicare item codes outlined in **Supplementary Table 1**. The postcode-derived Index of Relative Socio-Economic Advantage and Disadvantage, was used to define level of social advantage and divided into five predetermined strata, whereby a higher stratum indicates greater socioeconomic advantage. Being able to walk on admission for the index stroke event was used as a proxy measure for stroke severity and infers mild stroke [13].

Comorbidities were identified based on medications dispensed in the year prior to stroke using relevant pharmaceutical claim item codes and grouped as 0, 1, 2, or ≥3 [14]. Registrants claiming for services from a Primary Care Practitioner and at least two types of allied health professionals were identified as receiving multidisciplinary care.

### 2.4 Statistical Analyses

Descriptive statistics are reported to describe characteristics of the AuSCR registrants. Latent Class Analysis (LCA) was used to classify registrants into ‘classes’ based on patterns of responses across the five domains of the EQ-5D-3L. LCA classes are identified algorithmically based on similar response profiles, which we hypothesized would reflect varying degrees of impairment. The LCA approach allows for the investigation of an unobservable categorical latent variable (here, ‘impairment post-stroke’) based on multiple observed categorical variables known as indicators (here, the EQ-5D-3L domains). For LCA, the ‘optimum’ model should represent a meaningful and conceptually useful set of groupings [15]. We explored models ranging from 2–5 classes, and with the EQ-5D-3L as a two-level indicator in which “some problems”, and “extreme problems” were collapsed into a single category, and as a three-level indicator. We computed the Akaike (AIC) and Bayesian information criterions for all candidate models which converged. Each candidate model was examined for plausibility, utility, and size of individual clusters to inform final choice.

Five multivariable logistic regression models were constructed to assess associations between impairment (LCA class) and having a claim for: (i) a chronic disease management plan or review, (ii) multidisciplinary care coordination or review, (iii) Medicare subsidised allied health services, and (iv) multidisciplinary care (as defined above), within 18 months following stroke. All models were adjusted for age, sex, stroke severity, stroke type and prior stroke, derived from the AuSCR, as well as comorbidities (pharmaceutical claim item codes), total number of Primary Care Practitioners encounters and specialist involvement. To assess the effect of survivor bias, a sensitivity analysis restricting the cohort to those who survived to 18 months after admission for stroke was performed. Analyses were conducted using Stata MP 15.0 (StataCorp, Texas, TX, USA) and model performance tested using the Hosmer-Lemeshow goodness of fit test [16].

## 3. Results

**Fig. 1.**
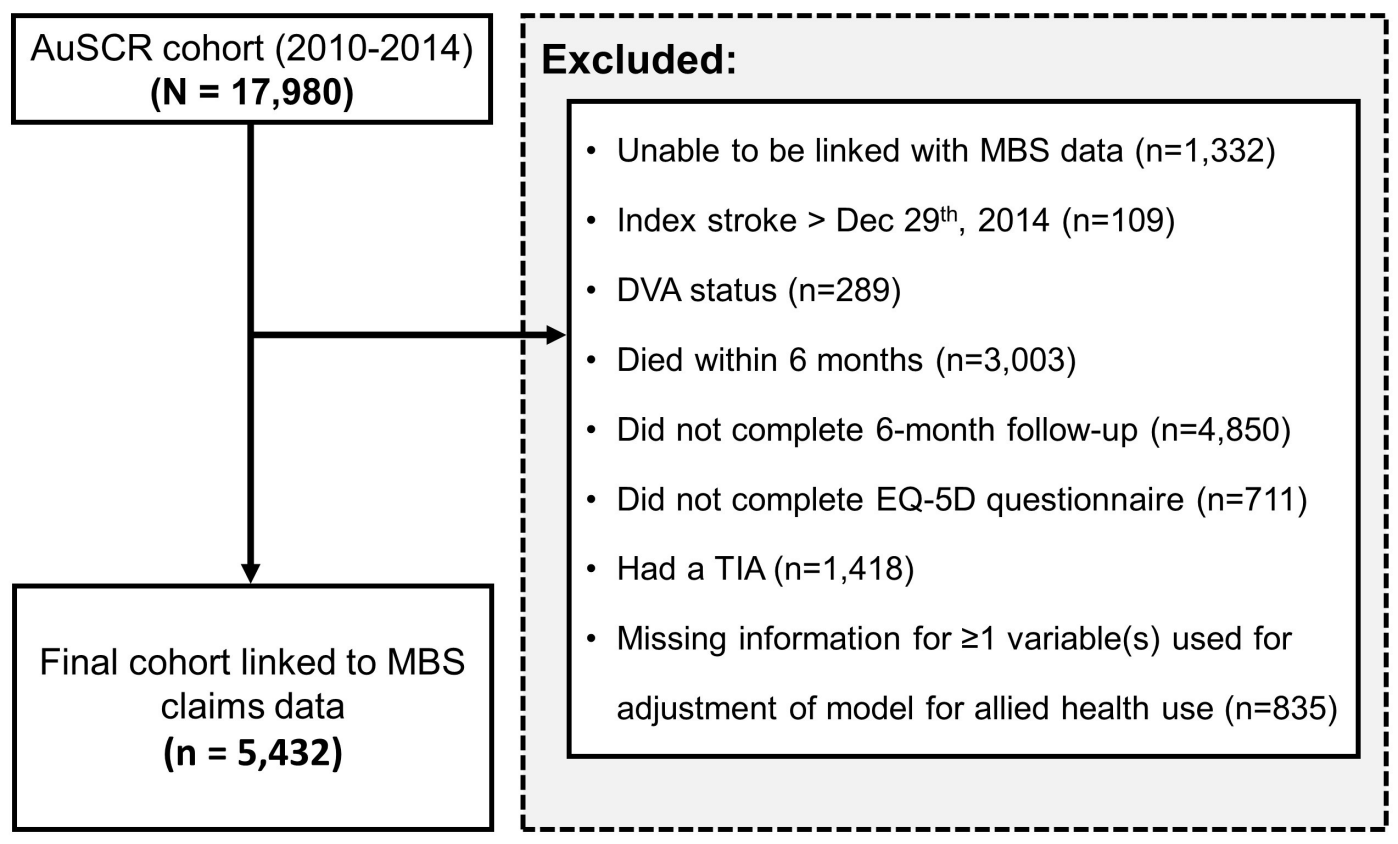
**Flow diagram of participants included in our latent class analysis**. AuSCR, Australian Stroke Clinical Registry; MBS, Medicare Benefit Schedule; DVA, Department of Veterans Affairs; TIA, Transient Ischaemic Attack.

Of the initial AuSCR cohort (N = 17,980), 93% were linked with Medicare and 5432 were eligible for inclusion in analysis (Fig. 1). Of these, 72% were aged ≥65 years, 44% were female, 86% had experienced ischaemic stroke, and 57% had severe stroke (Table 1). AuSCR registrants who complete the follow-up survey have been shown to be demographically and clinically similar to non-responders [17].

**Table 1.** **Overall cohort of responders and patients assigned to three impairment classes**.

Patient characteristics		Total	Severity of impairment:			*p*-value
		n (%)†	Minimal	Moderate	Severe	
		n = 5432	n = 2576	n = 2293	n = 563	
			(47.4%)	(42.2%)	(10.4%)	
EQ-5D responses, across all domains						
	0 problems in any domain	1318 (24.3)	1318 (51.1)	0	0	
	1 problem	760 (14.0)	760 (29.5)	0	0	
	2 problems	707 (13.0)	498 (19.3)	209 (9.1)	0	
	3 problems	812 (15.0)	0	762 (33.2)	50 (8.9)	
	4 problems	867 (15.9)	0	743 (32.4)	124 (22.0)	
	5 problems (all domains impacted)	968 (17.8)	0	579 (25.3)	389 (69.1)	<0.001
EQ-5D Visual Analogue Scale (0–100), median (Q1, Q3)		70 (50, 85)	80 (75, 90)	60 (50, 72)	40 (25, 50)	<0.001
Died (6–18 months post-stroke)		351 (6.5)	61 (2.4)	167 (7.3)	123 (21.9)	<0.001
Age, years						
	18–64	1536 (28.3)	904 (35.1)	561 (24.5)	71 (12.6)	
	65–74	1374 (25.3)	762 (29.6)	509 (22.2)	103 (18.3)	
	75–84	1763 (32.5)	727 (28.2)	804 (35.1)	232 (41.2)	
	≥85	759 (14.0)	183 (7.1)	419 (18.3)	157 (27.9)	<0.001
Female		2365 (43.5)	988 (38.4)	1060 (46.2)	317 (56.3)	<0.001
Severe stroke‡		3114 (57.3)	1120 (43.5)	1507 (65.7)	487 (86.5)	<0.001
Type of stroke						
	Intracerebral haemorrhage	604 (11.1)	237 (9.2)	270 (11.8)	97 (17.2)	
	Ischaemic stroke	4695 (86.4)	2271 (88.2)	1969 (85.9)	455 (80.8)	
	Undetermined stroke	133 (2.5)	68 (2.6)	54 (2.4)	11 (2.0)	<0.001
	Previous history of stroke	227 (4.2)	62 (2.4)	124 (5.4)	41 (7.3)	<0.001
Number of comorbidities§						
	0	1722 (31.7)	926 (36.0)	579 (25.3)	217 (38.5)	
	1	1335 (24.6)	748 (29.0)	496 (21.6)	91 (16.2)	
	2	1141 (21.0)	503 (19.5)	536 (23.4)	102 (18.1)	
	≥3	1234 (22.7)	399 (15.5)	682 (29.7)	153 (27.2)	<0.001
Socioeconomic position						
	Q1, Most disadvantaged	642 (11.8)	280 (10.9)	295 (12.9)	67 (11.9)	
	Q2	884 (16.3)	406 (15.8)	382 (16.7)	96 (17.1)	
	Q3	1138 (21.0)	539 (20.9)	480 (20.9)	119 (21.1)	
	Q4	1269 (23.4)	588 (22.8)	560 (24.4)	121 (21.5)	
	Q5, Least disadvantaged	1499 (27.6)	763 (29.6)	576 (25.1)	160 (28.4)	0.04
Year of admission						
	2010	520 (9.6)	232 (9.0)	211 (9.2)	77 (13.7)	
	2011	584 (10.8)	265 (10.3)	230 (10.0)	89 (15.8)	
	2012	1115 (20.5)	540 (21.0)	472 (20.6)	103 (18.3)	
	2013	1487 (27.4)	698 (27.1)	659 (28.7)	130 (23.1)	
	2014	1726 (31.8)	841 (32.7)	721 (31.4)	164 (29.1)	<0.001

† Kruskal-Wallis H test comparison of mean ranks. ‡ Inability to walk on admission denotes severe stroke. § Based on pharmaceutical claim item codes from the Rx-Risk Comorbidity Index. Q1–Q5, quintiles of socioeconomic disadvantage, derived using the Index of Relative Socio-Economic Advantage and Disadvantage.

More than 99% of the study cohort saw a Primary Care Practitioners within 18 months of stroke and 13% had ≥1 claim for a medical specialist. During the study period 39% had a claim for a chronic disease management plan or review, 39% had a claim for multidisciplinary care coordination or review and 46% had a claim for Medicare-subsidised allied health care. Of those who claimed Medicare-funded allied health services, 47% claimed podiatry, 20% claimed physiotherapy services and 4% had a claim for services relevant to secondary prevention (exercise physiology or dietetics). Other types of allied health services such as speech pathology or occupational therapy were claimed by less than 2%.

For the LCA, two models converged, a model with: (1) two-level EQ-5D-3L indicators (none vs moderate/severe) and two classes, and (2) three-level indicators (none, moderate, severe) and three classes (**Supplementary Table 2**). The two-class model demonstrated a slightly lower AIC (29,462) than the three-class model (38,506). However, the three-class model was chosen as it produced groupings of patients with impairment that were clinically meaningful. Guided by the pattern in which patients within each class reported their impairment within each EQ-5D-3L domain (Table 1), we defined class 1 as patients with minimal impairment (n = 2576), class 2 as moderate impairment (n = 2293) and class 3 as severe impairment (n = 563). For example, those who indicated on the EQ-5D that they had no problems with mobility had a 99.6% odds of being allocated to the minimal impairment group whereas those confined to bed had a 100% odds of being allocated to the severe impairment group (**Supplementary Table 3**). An increase in age, stroke severity and number of comorbidities was also observed with increasing severity (Table 1).

**Fig. 2.**
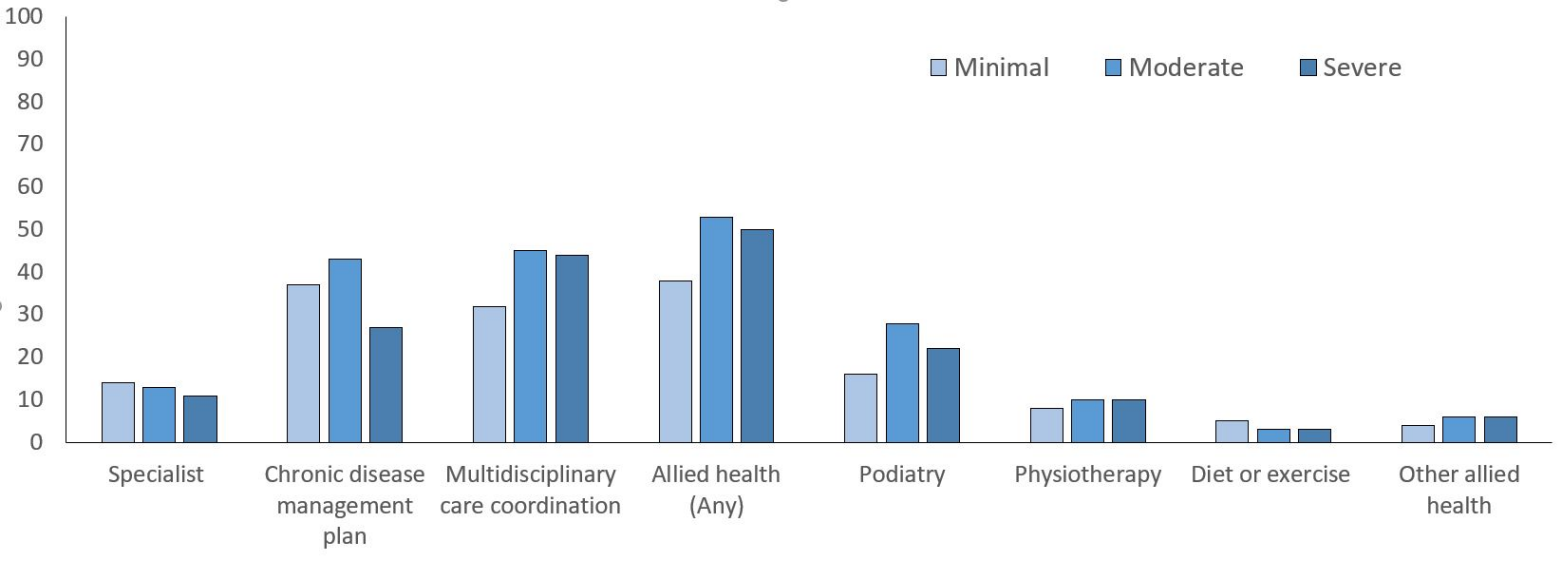
**Proportion (%) of cohort with Medicare claim items for Medicare funded enhanced primary care by impairment group**.

A smaller proportion of those with severe impairment claimed a chronic disease management plan or review in the 18 months following stroke (27%) than those with minimal (37%) or moderate (43%) impairment (Table 2, Fig. 2). Those with minimal impairment were less likely to have a claim for multidisciplinary care coordination or review than those with moderate or severe impairment (32% vs 45%, 44%). Podiatry claims were more common in those with moderate impairment (28%) than other classes (16% minimal, 22% severe). Few differences were observed for other allied health services except for non-pharmacological secondary prevention (dietetics or exercise physiology), where a greater proportion of those with minimal impairment had a claim for these services compared to those in the other two impairment groups (Table 2, Fig. 2).

**Table 2.** **Medicare claim items for enhanced models of primary care in the 18 months following stroke, by impairment group**.

Medicare claims	Total	Severity of impairment:			*p*-value
	n (%)†	Minimal	Moderate	Severe	
	n = 5432	n = 2576	n = 2293	n = 563	
		(47.4%)	(42.2%)	(10.4%)	
Medicare funded enhanced primary care plans					
Chronic disease management plan‡	2009 (38.6)	954 (37.0)	991 (43.2)	154 (27.4)	<0.001
Multidisciplinary care coordination‡	2116 (39.0)	833 (32.3)	1035 (45.1)	248 (44.1)	<0.001
Medicare funded allied health claims					
Allied health (Any)	2470 (45.5)	978 (38.0)	1208 (52.7)	284 (50.4)	<0.001
Podiatry	1173 (21.6)	415 (16.1)	636 (27.7)	122 (21.7)	<0.001
Physiotherapy	501 (9.2)	210 (8.2)	235 (10.3)	56 (10.0)	0.03
Dietetics service or exercise physiology	214 (3.9)	123 (4.8)	75 (3.3)	16 (2.8)	0.01
Occupational Therapy§	18 (0.3)	≤8 (≤0.3%)	≤8 (≤0.3%)	≤8 (≤1.4%)	0.68
Speech Therapy	41 (0.8)	17 (0.7)	17 (0.7)	7 (1.2)	0.35
Other allied health	138 (3.6)	64 (3.4)	66 (3.9)	8 (3.0)	0.14

† Kruskal-Wallis H test comparison of mean ranks. ‡ includes reviews. § Actual cell counts cannot be reported due to ethical restrictions that prevent any cell from reporting <5 individuals.

In multivariable logistic regression, those with moderate impairment were more likely to claim a chronic disease management plan or review (adjusted Odds Ratio (aOR): 1.24, 95% Confidence Interval (CI): 1.10, 1.40) than those with minimal impairment (Table 3). However, those with severe impairment were almost 40% less likely to claim this care type (aOR: 0.61, 95% CI: 0.49, 0.75). Those with moderate (aOR 1.53, 95% CI 1.35, 1.73) or severe impairment (aOR 1.49, 95% CI: 1.23, 1.81) were more likely to claim multidisciplinary care coordination or review than those with minimal impairment. Results for allied health care were similar to those for multidisciplinary care coordination. The overall results remained similar when analyses were restricted to those surviving 18 months post-stroke (**Supplementary Table 4**).

**Table 3.** **Univariable and multivariable logistic regression of association between level of impairment and health service claims**.

Models (n = 5432)	Univariable	*p*-value	Multivariable	*p*-value
	OR (95% CI)		OR (95% CI)	
Chronic disease management plan	Reference group: minimal impairment			
Moderate impairment	1.30 (1.16, 1.46)	<0.001	1.25 (1.10, 1.41)	<0.001
Severe impairment	0.60 (0.49, 0.72)	<0.001	0.61 (0.49, 0.75)	<0.001
Multidisciplinary care coordination	Reference group: minimal impairment			
Moderate impairment	1.72 (1.53, 1.93)	<0.001	1.53 (1.35, 1.73)	<0.001
Severe impairment	1.65 (1.37, 1.98)	<0.001	1.50 (1.23, 1.83)	<0.001
Allied health services†	Reference group: minimal impairment			
Moderate	1.82 (1.62, 2.04)	<0.001	1.56 (1.38, 1.76)	<0.001
Severe	1.66 (1.38, 2.00)	<0.001	1.41 (1.16, 1.72)	<0.01
Multidisciplinary Care‡	Reference group: minimal impairment			
Moderate	1.29 (1.03, 1.67)	0.048	1.30 (0.99, 1.70)	0.06
Severe	1.10 (0.72, 1.68)	0.659	1.28 (0.81, 2.00)	0.29

OR, odds ratio; CI, confidence interval. All models were adjusted for age group, type of stroke, sex, inability to walk on admission (indicative of stroke severity), previous stroke, number of comorbidities, specialist claim (including psychiatry) and socioeconomic status. † Services includes those funded through Medicare: physiotherapy, podiatry, exercise physiology, dietetics, audiology, chiropractor, diabetes education, mental health worker, osteopathy and psychology. ‡ Claimed services from a primary care practitioner and at least two types of allied health professionals under a multidisciplinary care coordination plan.

## 4. Discussion

Using population linked data, we have identified important gaps in how Medicare funded enhanced primary care policies are used to support people living with stroke. Only 39% of people living with stroke had a Medicare claim for chronic disease management or multidisciplinary care coordination in the 18 months following stroke. Those with greatest impairment, who are likely to benefit most, were less likely to have claimed a chronic disease management plan than those with lesser impairments. There was little variation in claims for Medicare subsidised allied health services based on impairment, with claims predominantly used for podiatry, indicating that these items are not being optimally prescribed. The exception being allied health claims for secondary prevention services which were most likely to be accessed by those with minimal impairment.

Enhanced primary care policies have the potential to comprehensively support people with stroke to be managed according to clinical guidelines [6]. This potential benefit is supported by evidence of improved adherence to recommended care for diabetes [18] and reduced hospital readmissions for heart failure in Veteran populations [19]. Despite these potential benefits, our results highlight that Medicare incentivised policies directed at enhanced primary care are underutilised in people living with stroke. It is also concerning that claims for chronic disease management plans varied by impairment group with those with severe impairment being most disadvantaged. This is particularly concerning given the greater prevalence of cardiovascular disease in those with disability or frailty [20, 21]. Sub-optimal management of those with impairment may contribute to the increase in hospitalisation rates, as observed in a prior study, compared to those without impairment [17]. We provide preliminary evidence of gaps in primary care following stroke which may contribute to variability in health outcomes and inequity in access to recommended care.

According to clinical guidelines people with chronic impairments following stroke should be reviewed annually, and if identified as requiring further rehabilitation be referred to therapy services to set new goals and improve function [6]. In Australia, where annual follow-up with a stroke specialist is not standard practice, multidisciplinary care coordination provides an opportunity for Primary Care Practitioners to perform this role. These arrangements have been associated with greater self-assessed quality of care [22] and can support these processes to improve outcomes for people living with long-term impairment [23, 24]. The greater rates of use in those with moderate and severe impairment indicates that these Medicare items are generally being claimed appropriately, despite the overall uptake being sub-optimal.

Multidisciplinary care coordination plans only allow claims for five visits per year across all eligible allied health services [8]. Nevertheless, there is potential for these to enhance or subsidise allied health services provided through other avenues such as private insurance or community health. Consideration should be given by Primary Care Practitioners and patients as to which allied health services are most appropriate to address the patient’s health and impairment needs. This is important because referral to the most appropriate profession can lead to better patient outcomes, including reduced preventable hospital readmissions [25]. In our study, the most commonly claimed profession was podiatry, being more than double the second most commonly claimed profession, physiotherapy. While this finding is consistent with previous evaluations in older Australian populations, the lack of diversity in allied health prescribing is concerning [25]. In prior Australian surveys 84% of people living with stroke reported a range of unmet health needs [1, 2] indicating the need for improved access to allied health care that addresses stroke related impairments. Although it is possible that registrants were accessing allied health services through other avenues not captured in Medicare claims, our results suggest that Medicare-subsidised allied health items are not being optimally used to meet the needs of people living with stroke.

In addition to facilitating ongoing rehabilitation following stroke, the multidisciplinary care coordination plan can be used to refer for non-pharmacological management of risk factors for recurrent stroke such as high cholesterol, high blood pressure and physical inactivity [26]. Guidelines recommend that all people living stroke should receive non-pharmacological management of risk factors, including advice and support on maintaining a healthy diet and recommended physical activity levels [6]. Similar to variations in access to chronic disease management plans, those with severe impairment were less likely to claim for dietetic and exercise physiology services than those with mild or moderate impairment, further disadvantaging this group with regards to secondary prevention management. Again, this suggests potential biases or disproportionate gaps in secondary prevention management in those with severe impairments.

The strengths of this study include a large nationally representative sample of people with stroke from urban and rural locations with collection of quality of life outcome data to classify impairment at 90–180 days post stroke. The availability of personal information (name, surname, date of birth) from AuSCR supported person-level linkages with Medicare claims data for reliable exposure classification. The main limitation is that our findings are restricted to services claimed through Medicare. Some of the variability in care observed in those with severe impairment may be due to a greater probability of people within our Medicare group receiving end of life care. However, this is likely to account for only a small proportion of our cohort as we excluded those residing in residential care facilities.

## 5. Conclusions

The use of Medicare funded policies has potential to support Primary Care Practitioners to manage people with stroke according to clinical guidelines and fill gaps in long-term care for those with ongoing impairments. Despite this, uptake is poor for those living with stroke and variability exists with regards to how these items are prescribed. As such, it is unlikely that these models of care are being optimally used to address the ongoing rehabilitation needs of people with stroke, or assist in the prevention of secondary stroke. Further work is underway, through the PRECISE study [27] to determine the impacts on long-term patient outcomes, with potential to modify uptake and facilitate expansion of these programs by government to better address the needs of people living with stroke.

## Supplementary Material

Supplementary material associated with this article can be found, in the online version, at https://doi.org/10.31083/j.rcm2309318.
