# Supplementary material for: Are Medicare Funded Multidisciplinary Care Policies Being Claimed in accordance to Rehabilitation Needs in Patients with Stroke?
Source: Rev Cardiovasc Med. 2022 Sep 14;23(9):318. doi: 10.31083/j.rcm2309318 (PMC11262366; doi:10.31083/j.rcm2309318)
Supplement: Supplementary file 1 [file 2153-8174-23-9-318-s1.docx]

Supplementary Table 1. Medicare Benefits Schedule item codes used to identify receipt of a chronic disease management plan, multidisciplinary care coordination or allied health care within first 18 months of stroke.

| Type of visit | MBS item codes |
| --- | --- |
| Chronic disease management plan or review | 721, 732 |
| Multidisciplinary care coordination or review | 723, 731 |
| Allied health care | Any of the codes below |
| Audiology | 10952 |
| Chiropractor | 10964 |
| Diabetes education service | 10951 |
| Dietetics services | 10954 |
| Exercise physiology | 10953 |
| Mental health service | 10956 |
| Occupational therapy | 10958 |
| Osteopathy | 10966 |
| Physiotherapy | 10960 |
| Podiatry | 10962 |
| Psychiatry | 228 to 370 |
| Psychology | 10968 |
| Speech pathology | 10970 |
| Specialist | 99, 104, 105, 107, 108, 110, 112, 114, 116, 117, 119, 120, 122, 128, 131, 132, 133, 141, 142, 143, 144, 145, 146, 147, 149 |

Supplementary Table 2. Comparison of possible latent class models.

| Model | Converge | Classes | Levels | AIC | BIC |
| --- | --- | --- | --- | --- | --- |
| 1 | Yes | 1 | 2 | 38,507 | 38,573 |
| 2 | Yes | 1 | 3 | 48,188 | 48,254 |
| 3 | Yes | 2 | 2 | 29,462 | 29,535 |
| 4 | No convergence | 2 | 3 | - | - |
| 5 | No convergence | 3 | 2 | - | - |
| 6 | Yes | 3 | 3 | 38,506 | 38,717 |
| 7 | No convergence | 4 | 2 | - | - |
| 8 | No convergence | 4 | 3 | - | - |

AIC: Akaike information criterion, BIC: Bayesian information criterion.

Supplementary Table 3. Median estimated probabilities for class membership given each level of indicator that comprises the European Quality of Life Scale (EQ-5D).

|  | Total (n=5,432)  Probability (95% CI) | | |
| --- | --- | --- | --- |
| Indicator | Class 1 n=2,576 | Class 2 n=2,293 | Class 3 n=563 |
| **Mobility** |  |  |  |
| 0 – No problems in walking about | 99.6 (92.1, 99.9) | 0.4 (0.1, 7.9) | - |
| 1 – Some problems | 0.1 (0.0, 2.5) | 97.5 (54.0, 99.6) | 0.1 (0.0, 14.8) |
| 2 – Confined to bed | - | - | 100.0 (100, 100) |
| **Self care** |  |  |  |
| 0 – No problems | 96.9 (20.9, 99.9) | 3.1 (0.1, 79.1) | - |
| 1 – Some problems washing/dressing self | - | 99.6 (62.4, 99.7) | 0.4 (0.2, 31.1) |
| 2 – Unable to wash/dress self | - | 0.0 (0.0, 1.8) | 100.0 (98.2, 100.0) |
| **Usual activity** |  |  |  |
| 0 – No problems | 99.9 (99.2, 99.9) | 0.1 (0.1, 0.8) | - |
| 1 – Some problems | 0.5 (0.0, 20.9) | 98.8 (79.1, 99.6) | - |
| 2 – Unable to perform | - | 27.2 (0.0, 62.4) | 72.8 (37.6, 100.0) |
| **Pain/discomfort** |  |  |  |
| 0 – No pain/discomfort | 99.6 (54.4, 99.9) | 0.1 (0.1, 31.6) | - |
| 1 – Moderate pain/discomfort | 0.5 (0.0, 17.1) | 82.9 (3.9, 99.5) | - |
| 2 – Extreme pain/discomfort | 0.0 (0.0, 0.2) | 27.2 (0.2, 98.8) | 5.4 (0.0, 99.8) |
| **Anxiety/depression** |  |  |  |
| 0 – Not anxious/depressed | 99.2 (2.5, 99.9) | 0.8 (0.1, 75.8) | - |
| 1 – Moderately anxious/depressed | 0.5 (0.0, 55.8) | 64.2 (3.9, 99.5) | 0.0 (0.0, 2.4) |
| 2 – Extremely anxious/depressed | 0.0 (0.0, 0.3) | 27.4 (0.2, 98.8) | 3.5 (0.0, 99.8) |

Estimated probabilities are reported as median % (Q_1_, Q_3_).

Empty cell indicates when estimated median (Q_1_, Q_3_) probabilities were less than 0.04.

Values of ‘100.0’ were rounded up for values 99.500-99.999.

Supplementary Table 4. Univariable and multivariable logistic regression of association between level of impairment and use of health services among those who survived to 18 months post discharge

|  | Univariable |  | Multivariable |  |
| --- | --- | --- | --- | --- |
| Models | OR (95% CI) | P‑value | OR (95% CI) | *P*‑value |
| **Chronic disease**  *reference: minimal impairment*  **management plan** | | | | |
| Moderate  Severe | 1.36 (1.21, 1.53)  0.69 (0.56, 0.85) | <0.001  <0.001 | 1.28 (1.13, 1.45)  0.64 (0.51, 0.81) | <0.001  <0.001 |
| **Multidisciplinary care** *reference: minimal impairment*  **coordination** | | | | |
| Moderate  Severe | 1.77 (1.57, 2.0)  1.88 (1.53, 2.30) | <0.001  <0.001 | 1.55 (1.37, 1.76)  1.59 (1.28, 1.98) | <0.001  <0.001 |
| **Allied Health services†**  *reference:* *minimal impairment* | | | | |
| Moderate  Severe | 1.87 (1.66, 2.10)  1.82 (1.48, 2.23) | <0.001  <0.001 | 1.58 (1.39, 1.79)  1.45 (1.17, 1.80) | <0.001  <0.01 |
| **Multidisciplinary care‡**  *reference minimal impairment* | | | | |
| Moderate  Severe | 1.38 (1.07, 1.78)  1.30 (0.84, 2.01) | 0.01  0.3 | 1.37 (1.05, 1.80)  1.39 (0.87,2.21) | 0.02  0.2 |

OR, odds ratio; CI, confidence interval; GP, general practitioner.

All models were adjusted for age group, type of stroke, sex, inability to walk on admission (indicative of stroke severity), previous stroke, number of comorbidities, specialist claim (including psychiatry) and socioeconomic status.

**†** Services includes those funded through Medicare: physiotherapy, podiatry, exercise physiology, dietetics, audiology, chiropractor, diabetes education, mental health worker, osteopathy and psychology.
‡ Claimed services from a general practitioner and at least two types of allied health professionals under a multidisciplinary care coordination plan.
